# The relationships between cholesterol crystals, NLRP3 inflammasome, and coronary atherosclerotic plaque vulnerability in acute coronary syndrome: An optical coherence tomography study

**DOI:** 10.3389/fcvm.2022.905363

**Published:** 2022-10-25

**Authors:** Chao Xue, Qizhi Chen, Ling Bian, Zhaofang Yin, Zuojun Xu, Huili Zhang, Qingyong Zhang, Junfeng Zhang, Changqian Wang, Run Du, Li Fan

**Affiliations:** ^1^Department of Cardiology, Ninth People's Hospital, Shanghai Jiao Tong University School of Medicine, Shanghai, China; ^2^Department of Cardiovascular Medicine, Ruijin Hospital, Shanghai Jiao Tong University School of Medicine, Shanghai, China

**Keywords:** acute coronary syndrome, cholesterol crystals, NLRP3 inflammasome, interleukin (IL)-1β, IL-18, optical coherence tomography, vulnerable plaque

## Abstract

**Background:**

Cholesterol crystals (CCs) in lesions are the hallmark of advanced atherosclerotic plaque. Previous studies have demonstrated that CCs could activate NLRP3 inflammasome, which played an important role in atherosclerotic lesion progression. However, the relationship between CCs, NLRP3 inflammasome pathway, and plaque vulnerability in patients with ACS is still not elucidated.

**Methods:**

Two hundred sixty-nine consecutive acute coronary syndrome (ACS) patients with 269 culprit lesions were included in this study. CCs and other plaque characteristics within the culprit lesion segment were evaluated by optical coherence tomography (OCT) before percutaneous coronary intervention (PCI). The NLRP3 mRNA expression in peripheral blood mononuclear cells (PBMCs) and the serum levels of interleukin (IL)-1β, IL-18, and other biological indices were measured.

**Results:**

Cholesterol crystals were observed in 105 (39%) patients with 105 culprit lesions. There were no significant differences in baseline clinical characteristics between the patients with CCs (CCs group, *n* = 105) and the patients without CCs (non-CCs group, *n* = 164) within the culprit lesion segment except for lipoprotein(a) [Lp(a)]. The CCs group had a higher level of NLRP3 mRNA expression in PBMCs and higher levels of serum cytokine IL-1β and IL-18. OCT showed that the CCs group had longer lesion length, more severe diameter stenosis, and less minimum luminal area (MLA) than the non-CCs group (all *p* < 0.05). The frequency of thin-cap fibroatheroma (TCFA), thrombus, accumulation of macrophages, plaque rupture, micro-channel, calcification, spotty calcification, and layered plaque was higher in the CCs group than in the non-CCs groups (all *p* < 0.05). Multivariate logistic analysis revealed that the level of NLRP3 expression (OR = 10.204), IL-1β levels (OR = 3.523), IL-18 levels (OR = 1.006), TCFA (OR = 3.593), layered plaque (OR = 5.287), MLA (OR = 1.475), macrophage accumulation (OR = 2.881), and micro-channel (OR = 3.185) were independently associated with CCs.

**Conclusion:**

Acute coronary syndrome patients with CCs in culprit lesions had a higher expression of NLRP3, IL-1β, and IL-18, and had more vulnerable plaque characteristics than patients without CCs. CCs might have interacted with NLRP3 inflammasome activation in patients with ACS, which could contribute to plaque vulnerability in culprit lesions.

## Introduction

Despite tremendous achievements in its management, acute coronary syndrome (ACS) remains the leading cause of death worldwide (1). The fundamental pathology of ACS is the rupture or erosion of vulnerable plaque and subsequent thrombus formation (2, 3). Therefore, the identification of vulnerable plaque before its rupture is a pivotal measure to prevent ACS. Optical coherence tomography (OCT) is a novel high-resolution intracoronary imaging technology that allows the resolution of tissue microstructural interfaces ranging from 10 to 20 μm (4). It has been reported that OCT enables *in vivo* visualization of various lesion features associated with plaque vulnerability such as thin-cap fibroatheroma (TCFA), plaque rupture, plaque erosion, macrophage accumulation, intracoronary thrombus, intimal vasculature, calcification, and cholesterol crystals (CCs) (5). The presence of high-risk OCT plaque features was found to be associated with a higher risk of major coronary events (6). The images of CCs in OCT are thin, linear, sharp-bordered regions with high intensity (7). Previous studies have demonstrated that CCs were frequently found in human atherosclerotic plaques of different stages from fatty streaks to advanced lesions (8). Recently, some studies showed that CCs were associated with vulnerable plaque morphological features (9–11). However, the mechanisms of CCs-inducing plaque destabilization were not fully elucidated.

Nucleotide-binding domain leucine-rich repeat-containing (NLR) family, pyrin domain containing 3 (NLRP3) is among the family members of NOD-like receptors (NLRs). Upon activation, the NLRP3 recruits the adapter protein and forms the NLRP3 inflammasome, a macromolecular protein complex that leads to the activation of caspase-1 and promotes the maturation and release of inflammatory cytokines interleukin (IL)-1β and IL-18 (12). Previous animal and human studies had demonstrated the important role of NLPR3 in the severity and progression of coronary atherosclerosis (13–15). Pathohistological studies demonstrated that the NLRP3 inflammasome could be activated by CCs, contributing to atherosclerotic lesion progression and its subsequent complications (16).

In this study, we aimed to explore the correlation between the CCs, plaque vulnerability, and NLRP3 inflammasome in culprit lesions in patients with ACS.

## Methods

### Study design and study population

A total of 332 consecutive patients with ACS at Ninth People's Hospital, Shanghai Jiao Tong University School of Medicine between January 2018 and December 2019 were enrolled in this study. Culprit lesions of all the patients were examined by OCT prior to intervention. ACS consisted of unstable angina pectoris (UA), ST-elevation myocardial infarction (STEMI), and non–ST-elevation myocardial infarction (NSTEMI). UA was defined as new-onset angina, angina at rest within 2 weeks or crescendo angina without elevated cardiac-specific biomarker troponin I/T (TnI/T). NSTEMI was defined as ischemic symptoms with TnI/T elevated but no ST-segment elevation on the electrocardiograph (ECG). STEMI was defined as continuous ischemic symptoms with new elevated ST-segment or new left bundle branch block (LBBB) in ECG and Tn I/T in plasma. We excluded those with cardiogenic shock (*n* = 8), prior coronary artery bypass graft (CABG) (*n* = 12) or prior PCI (*n* = 25) in the culprit vessel, serious liver or renal dysfunction (*n* = 11), and serious diseases such as malignant tumors and autoimmune diseases (*n* = 7). Finally, a total of 269 patients were included in our study. To evaluate the expression of NLRP3 mRNA in peripheral blood monocytes (PBMCs), 105 patients admitted to our hospital for chest tightness but with no coronary atherosclerotic stenosis at coronary angiography (CAG) were included as the control group (non-coronary artery disease group).

### OCT imaging acquisition and analysis

**Figure 1:**
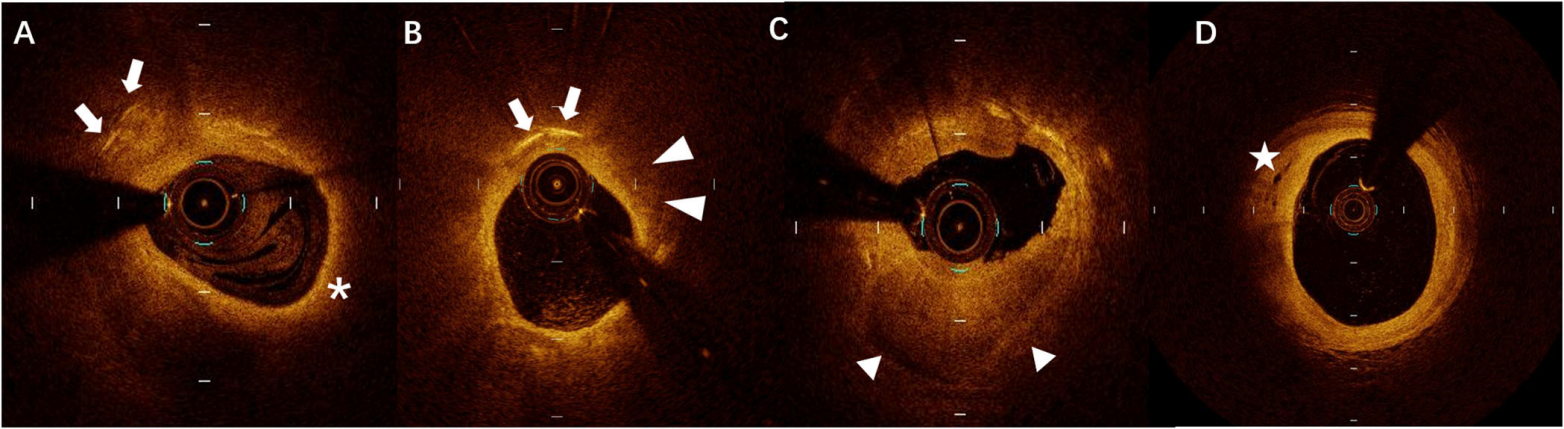
A representative case of optical coherence tomography (OCT) images of **(A)** cholesterol crystals (CCs) (arrows) and thin-cap fibroatheroma (TCFA) (asterisks), **(B)** CCs (arrows) and macrophages (arrowheads), **(C)** layered plaque (triangles), and **(D)** micro-channel (stars).

The culprit lesions were identified by the cardiologists based on ECG and CAG findings. Intracoronary OCT procedure was conducted for the culprit lesions after 100–200μg nitroglycerin was used before PCI. An aspiration catheter would be used for aspiration thrombectomy when there was insufficient antegrade coronary flow which made OCT imaging impossible. The acquisition of OCT images was achieved by using C7-XR OCT Intravascular Imaging System (St Jude Medical, St Paul, MN, USA). OCT images were analyzed by two independent investigators. When there was discordance between the observers, a consensus was obtained from a third independent investigator. Lipid length was recorded on a longitudinal view and max lipid arc was recorded. The presence of CCs, TCFA, ruptured plaque, erosion plaque, macrophages, micro-channel, thrombus, and spotty calcification in OCT was identified as described previously (11). Layered plaques in OCT were plaques with one or more layers of different optical densities (17). All typical images are shown in Figure 1.

### Biochemical measurements

Blood samples were collected after diagnosis and before PCI. Human peripheral blood monocytes (PBMCs) were isolated from venous blood by density gradient centrifuging and stratifying using a Ficoll-Paque Plus density gradient. TRIzol reagent (Invitrogen, Carlsbad, CA, USA) was used to isolate total RNA from PBMCs. The expression of ratios of NLRP3 mRNA was determined using the relative quantitative method with GAPDH as an internal control and fold changes were calculated. NLRP3 and GAPDH mRNA levels were measured as we described previously (18). The primer sequences used for qPCR are shown in Table 1. After collection, serum samples were stored at −80°C before being used. With specific enzyme-linked immunosorbent assay kits (R&D Systems, Minneapolis, MN, USA), serum levels of IL-1β and IL-18 were measured according to the manufacturer's guidance.

**Table 1:** Primer sequences.

NLRP3 (Human)	
Forward Primer	5^′^-GGTGTCTAGCATTGCGGTCA-3^′^
Reverse Primer	5^′^-TGCTCCTTGTCAGGGTTGAG-3^′^
GAPDH (Human)	
Forward Primer	5^′^-TGTGGGCATCAATGGATTTGG-3^′^
Reverse Primer	5^′^-ACACCATGTATTCCGGGTCAAT-3^′^

### Statistics

Statistical analysis was performed using SPSS (version 21, IBM Corp, Armonk, NY, USA). Continuous variables were expressed as mean ± standard deviation (SD). Independent samples Student's *t-*tests or Mann–Whitney *U*-tests were used for the comparison between CCs or non-CCs groups according to the data distribution. One-way ANOVA was used for comparison between patients with STEMI, NSTEMI, and UA. Categorical variables were presented as counts and frequencies and compared with the Chi-square test or Fisher's exact test. Multivariable logistic regression analysis with variables of clinical characteristics and OCT parameters was used to explore the independent risk factors of CCs. A *p-*value of <0.05 was considered statistically significant.

## Results

### Baseline characteristics

A total of 269 ACS patients with 269 culprit lesions were included in this study. Of them, 39% of patients with ACS (*n* = 105) were found to have CCs in culprit lesions. The baseline characteristics and angiographic features of the patients between the CCs group and the non-CCs group are shown in Table 2. No patient had a history of other vascular diseases including carotid artery disease, peripheral artery disease, or aortic aneurysm. There were no significant differences between the CCs group and the non-CCs group in age, gender, conventional atherosclerosis risk factors (smoking, hypertension, and diabetes mellitus), prior ACS, and clinical presentation (UA, NSTEMI, and STEMI). Both groups had similar culprit vessel distribution (*p* = 0.682). In addition, C reactive protein (CRP), brain natriuretic peptide (BNP), fasting blood glucose (FBG), hemoglobin A1c (HbA1C), total cholesterol (TC), triglycerides (TGs), low-density lipoprotein cholesterol (LDL-C), high-density lipoprotein cholesterol (HDL-C), apolipoprotein B (Apo B), apolipoprotein E (Apo E), TnI, creatine, uric acid, and left ventricular ejection fraction (LVEF) were similar between two groups. The level of Lp(a) (0.20 ± 0.21 g/L vs. 0.12 ± 0.13 g/L, *p* = 0.001) and proportion of elevated Lp(a) (>0.3g/L) (23.8 vs. 7.9%, *p* < 0.001) were much higher in the CCs group than that in the non-CCs group.

**Table 2:** Baseline characteristics.

**Characteristics**	**All subjects**	**CCs group**	**Non-CCs group**	***p*-value**
	**(*n =* 269)**	**(*n =* 105)**	**(*n =* 164)**	**CCs vs. non-CCs**
Age (years)	67.5 ± 10.3	66.7 ± 10.8	68.0 ± 9.8	0.320
Male (*n*, %)	201 (74.7)	78 (74.3)	123 (75.0)	0.887
Smoking (*n*, %)	90 (33.5)	34 (32.4)	56 (34.1)	0.792
Culprit vessel				
RCA (*n*, %)	72 (26.8)	32 (30.5)	40 (24.4)	0.210
LM (*n*, %)	2 (0.7)	0 (0)	2 (1.2)	0.522
LAD (*n*, %)	172 (63.9)	66 (62.8)	106 (64.6)	0.698
LCX (*n*, %)	23 (8.6)	7 (6.7)	16 (9.8)	0.504
Medical history				
Hypertension (*n*, %)	170 (63.2)	68 (64.8)	102 (62.2)	0.699
DM (*n*, %)	57 (21.2)	26 (24.8)	31 (18.9)	0.285
Dyslipidemia (*n*, %)	38 (14.1)	15 (14.3)	23 (14.0)	1.000
Prior ACS (*n*, %)	9 (3.3)	4 (3.8)	5 (3.0)	0.740
Presentation				0.113
UA (*n*, %)	109 (40.5)	35 (33.3)	74 (45.1)	
NSTEMI (*n*, %)	88 (32.7)	36 (34.3)	52 (31.7)	
STEMI (*n*, %)	72 (26.8)	34 (32.4)	38 (23.2)	
Laboratory data				
CRP (mg/l)	4.76 ± 11.64	5.70 ± 14.21	4.15 ± 9.65	0.329
BNP (pg/ml)	106.57 ± 217.48	139.03 ± 253.65	85.78 ± 188.70	0.066
FBG (mmol/l)	6.05 ± 2.11	6.26 ± 1.94	5.91 ± 2.21	0.182
HbA1C (%)	6.26 ± 1.55	6.26 ± 1.31	6.25 ± 1.69	0.987
D-Dimer (mg/l)	0.47 ± 0.54	0.44 ± 0.47	0.49 ± 0.58	0.512
TC (mmol/L)	4.09 ± 1.08	4.21 ± 0.97	4.00 ± 1.14	0.122
TG (mmol/L)	1.59 ± 1.02	1.60 ± 0.91	1.59 ± 1.09	0.928
HDL-C (mmol/L)	1.12 ± 0.62	1.16 ± 0.86	1.09 ± 0.40	0.430
LDL-C (mmol/L)	2.75 ± 1.07	2.90 ± 1.01	2.66 ± 1.10	0.066
Lp(a) (g/L)	0.15 ± 0.17	0.20 ± 0.21	0.12 ± 0.13	0.001
ApoB (g/L)	0.21 ± 0.13	0.23 ± 0.16	0.20 ± 0.12	0.139
ApoE (g/L)	3.935 ± 1.272	3.88 ± 1.23	3.97 ± 1.30	0.577
Lp(a) > 0.3g/L (*n*, %)	38 (14.1)	25 (23.8)	13 (7.9)	<0.001
Troponin I (ng/mL)	1.79 ± 10.04	3.19 ± 12.62	0.90 ± 7.89	0.098
Creatinine (umol/L)	88.52 ± 23.78	88.28 ± 23.87	88.13 ± 27.67	0.414
Uric acid (umol/L)	335.70 ± 95.71	342.37 ± 106.81	331.43 ± 87.96	0.361
LVEF (%)	59.03 ± 5.62	59.05 ± 5.57	59.02 ± 5.68	0.974

LAD, left anterior descending artery; RCA, right coronary artery; LCX, left circumflex artery; DM, diabetes mellitus; ACS, acute coronary syndrome; UA, unstable angina pectoris; NSTEMI, non–ST-elevation myocardial infarction; STEMI, ST-elevation myocardial infarction; CRP, C reactive protein; BNP, brain natriuretic peptide; FBG, fasting blood glucose; HbA1C, hemoglobin A1c; TC, total cholesterol; TG, triglycerides; HDL-C, high-density lipoprotein cholesterol; LDL-C, low-density lipoprotein cholesterol; Lp(a), lipoprotein(a); ApoB, apolipoprotein B; ApoE, apolipoprotein E; LVEF, left ventricular ejection fraction; CCs, cholesterol crystals.

### NLRP3 inflammasome and downstream cytokine expression

**Figure 2:**
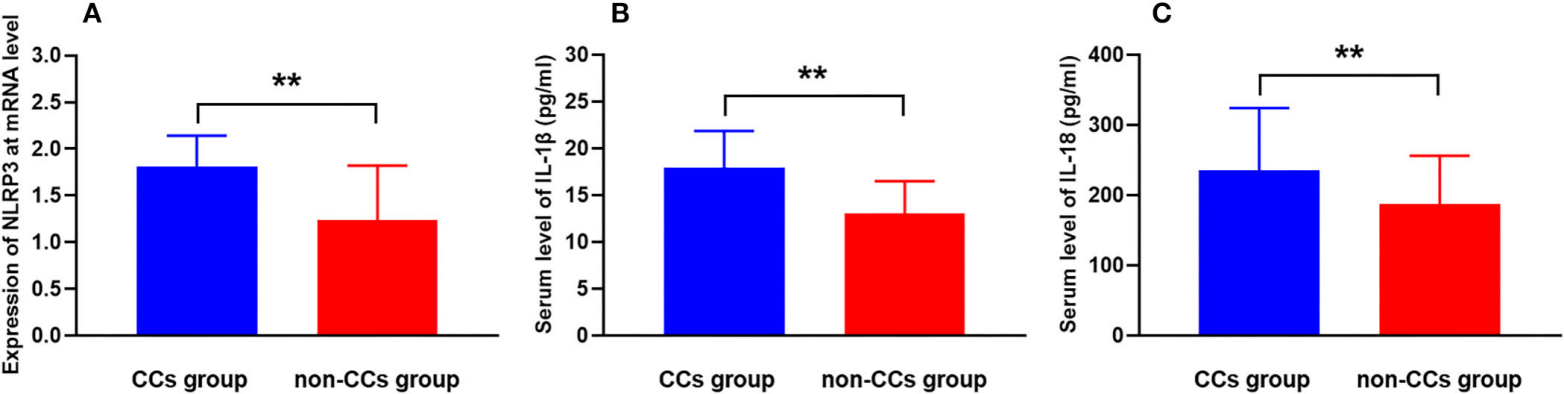
CCs in the plaque aggravate NLRP3 expression and inflammatory cytokine secretion in patients with ACS. Comparison of levels of **(A)** NLRP3 mRNA expression, **(B)** serum IL-1β, and **(C)** serum IL-18 between the CCs group and the non-CCs group in patients with ACS. **P < 0.01 vs. the non-CCs group.

The cholesterol crystals group had higher NLRP3 expression at the transcriptional level than the non-CCs group (1.81 ± 0.33 vs. 1.24 ± 0.58, *p* < 0.001) (Table 3, Figure 2A). Additionally, levels of serum IL-1β (17.95 ± 3.92 pg/ml vs. 13.06 ± 3.43 pg/ml, *p* < 0.001) and IL-18 (235.18 ± 88.98 pg/ml vs. 186.92 ± 68.99 pg/ml, *p* < 0.001) in patients with CCs were much higher than that in patients without CCs (Table 3, Figures 2B,C). There were no significant differences between patients with STEMI, NSTEMI, and UA with regard to the NLRP3 expression (1.55 ± 0.46 vs. 1.50 ± 0.62 vs. 1.37 ± 0.59, respectively, *p* = 0.075), level of serum IL-1β (15.66 ± 3.56 pg/ml vs. 14.95 ± 4.07 pg/ml vs. 14.43 ± 4.95 pg/ml, respectively, *p* = 0.176), and IL-18 (218.29 ± 74.50 pg/ml vs. 201.55 ± 92.34 pg/ml vs. 191.71 ± 73.14 pg/ml, respectively, *p* = 0.095).

**Table 3:** Levels of NLRP3 expression, serum IL-1β, and IL-18 in the CCs group and the non-CCs group.

**Expression**	**All subjects**	**CCs group**	**Non-CCs group**	***p*-value**
	**(*n =* 269)**	**(*n =* 105)**	**(*n =* 164)**	**CCs vs. non-CCs**
NLRP3 mRNA	1.46 ± 0.57	1.81 ± 0.33	1.24 ± 0.58	<0.001
IL-1β (pg/ml)	14.97 ± 4.34	17.95 ± 3.92	13.06 ± 3.43	<0.001
IL-18 (pg/ml)	205.76 ± 80.77	235.18 ± 88.98	186.92 ± 68.99	<0.001

CCs, Cholesterol crystals.

### Plaque characteristics assessed by OCT

The OCT findings of the culprit lesions are shown in Table 4. The lesion length (20.33 ± 9.36 mm vs. 16.48 ± 6.90 mm, *p* < 0.001) was much longer and diameter stenosis (DS) (68.92± 12.50% vs. 57.99 ± 17.0 %, *p* = 0.002) was more severe in the CCs group than that in the non-CCs group. The minimum luminal area (MLA), proximal luminal area (PLA), and distal luminal area (DLA) were much smaller in the CCs group than that in the non-CCs group (2.34 ± 1.29 mm^2^ vs. 3.63 ± 2.31 mm^2^, *p* < 0.001; 8.47 ± 2.74 mm^2^ vs. 9.47 ± 3.26 mm^2^, *p* = 0.010; 6.69 ± 2.86 mm^2^ vs. 7.46 ± 3.23 mm^2^, *p* = 0.046, respectively). The frequency of lipid-rich plaque and large calcification was similar between the two groups. However, lipid length (15.58 ± 9.98 mm vs. 12.38 ± 8.28 mm, *p* = 0.005) was much longer and maximal lipid arc was much larger (252.24 ± 110.16° vs. 206.50 ± 105.65°, *p* = 0.001) in the CCs group than that in the non-CCs group. In addition, TCFA (51.4 vs. 12.8%, *p* < 0.001), thrombus (37.1 vs. 16.4%, *p* = 0.026), macrophages accumulation (84.8 vs. 47.6%, *p* < 0.001), plaque rupture (24.8 vs. 7.3%, *p* < 0.001), micro channel (56.2 vs. 23.2%, *p* < 0.001), calcification (46.7 vs. 31.7%, *p* = 0.015), spotty calcification (44.8 vs. 29.9%, *p* = 0.019), and layered plaque (70.5 vs. 26.2%, *p* < 0.001) were more frequent in the CCs group than in the non-CCs group.

**Table 4:** Characteristics of OCT findings.

**Characteristics**	**All subjects**	**CCs group**	**Non-CCs group**	***p*-value**
	**(*n =* 269)**	**(*n =* 105)**	**(*n =* 164)**	**CCs vs. non-CCs**
MLA (mm^2^)	3.13 ± 2.07	2.34 ± 1.29	3.63 ± 2.31	< 0.001
PLA (mm^2^)	9.08 ± 3.10	8.47 ± 2.74	9.47 ± 3.26	0.010
DLA (mm^2^)	7.17 ± 3.11	6.69 ± 2.86	7.46 ± 3.23	0.046
ALA (mm^2^)	8.128 ± 2.89	7.58 ± 2.53	8.46 ± 3.06	0.014
Lesion length (mm)	17.98 ± 8.16	20.33 ± 9.36	16.48 ± 6.90	<0.001
DS (%)	62.26 ± 16.33	68.92 ± 12.50	57.99 ± 17.08	0.002
Lipid-rich plaque (n, %)	230 (85.5)	93 (88.6)	137 (83.5)	0.290
Lipid length (mm)	13.63 ± 9.10	15.58 ± 9.98	12.38 ± 8.28	0.005
Maximal lipid arc (°)	224.35 ± 109.53	252.24 ± 110.16	206.50 ± 105.65	0.001
TCFA (*n*, %)	75 (27.8)	54 (51.4)	21 (12.8)	< 0.001
Thrombus (*n*, %)	66 (24.5)	39 (37.1)	27 (16.4)	0.026
Macrophage (*n*, %)	167 (62.1)	89 (84.8)	78 (47.6)	<0.001
Plaque rupture (*n*, %)	38 (14.1)	26 (24.8)	12 (7.3)	<0.001
Micro channel (*n*, %)	97(36.0)	59(56.2)	38(23.2)	<0.001
Calcification (*n*, %)	101(37.5)	49(46.7)	52(31.7)	0.015
Spotty calcification (*n*, %)	96(35.7)	47(44.8)	49(29.9)	0.019
Large calcification (*n*, %)	56(20.8)	27(25.7)	29(17.7)	0.125
Layered plaque (*n* %)	117(43.5)	74(70.5)	43(26.2)	<0.001

MLA, minimum luminal area; PLA, proximal luminal area; DLA, distal luminal area; ALA, average luminal area; DS, diameter stenosis; TCFA, thin-capped fibroatheroma, CCs, cholesterol crystals.

### Predictors for cholesterol crystals

Multivariate logistic analysis revealed that the expression of NLRP3 (OR = 10.204; 95% *CI*: 4.546–22.904, *p* < 0.001), IL-18 (OR = 1.006; 95% *CI*: 1.002–1.011, *p* = 0.007), IL-1β (OR = 3.523; 95% *CI*: 1.743–8.875, *p* = 0.002), TCFA (OR = 3.593; 95% *CI*: 1.606–7.729, *p* = 0.038), layered plaque (OR = 5.287; 95% *CI*: 2.402–11.637, *p* < 0.001), MLA (OR = 1.475; 95% *CI*: 1.021–2.131, *p* = 0.039), macrophage accumulation (OR = 2.881; 95% *CI*: 1.179–7.0404, *p* = 0.020), and micro channel (OR = 3.185; 95% *CI*: 1.455–6.973, *p* = 0.004) were independently associated with CCs (Table 5).

**Table 5:** Multivariable logistic regression analysis for CCs.

**Variables**	**OR**	**95%CI**	***p*-value**
NLPR3 mRNA	10.204	4.546–22.904	<0.001
IL-18	1.006	1.002–1.011	0.007
IL-1β	3.523	1.743–8.875	0.002
TCFA	3.593	1.606–7.729	0.038
Layered plaque	5.287	2.402–11.637	<0.001
Macrophage	2.881	1.179–7.040	0.020
MLA	1.475	1.021–2.131	0.039
Micro channel	3.185	1.455–6.973	0.004

TCFA, thin-capped fibroatheroma; MLA, minimum luminal area; OR, odds ratio; CI, confidence interval.

## Discussion

The present study showed that CCs in culprit lesions were associated with vulnerable plaque features in patients with ACS. Furthermore, our study demonstrated for the first time the correlation between CCs in culprit lesions and NLRP3 pathway activation in the human body, which was only proved previously in cell and animal studies.

Studies concerning the formation of CCs have revealed that CCs were frequently found in atherosclerotic plaques and were present at all stages of atherogenesis (9, 19). An animal-based study found that CCs began to appear in the early stage of atherosclerotic plaque formation (20). An experiment with human artery samples demonstrated that CCs could be discovered from fatty streaks to advanced lesions (21). The incidence of CCs detected by intravascular imaging modalities or other imaging instruments in patients with coronary artery disease (CAD) varied in different studies. Nishimura et al. used OCT to identify the plaque components and found that 38% of patients with CAD (including ACS and stable angina pectoris, SAP) had CCs within the culprit lesion segment (10). Fujiyoshi et al. found that the incidence of CCs detected by OCT in culprit lesions requiring PCI was 29% (7). Tian et al. performed an OCT examination for all three coronary arteries in 255 patients and found that of 643 plaques detected, the frequency of CCs in severely stenotic TCFA was 40% (22). In a study evaluating aspirates for CCs by scanning electron microscopy (SEM) in patients with AMI who had the aspiration of culprit coronary artery obstruction, CCs were detected in 89% of all analyzed aspirates (23). Our study found that 39% of patients with ACS (including UA, NSTEMI, and STEMI) had CCs in culprit lesions.

The exact mechanism of CCs formation has not been clarified. The prevailing current view of CCs formation in atherosclerotic plaque is that macrophages and smooth muscle cells could convert the esterified cholesterol provided by the LDL located in sub-intima into free cholesterol. Free cholesterol was transported to high-density lipoprotein (HDL) through membrane-bound cholesterol carriers. Impaired HDL transport function or disequilibrium between esterified cholesterol and free cholesterol can lead to intracellular and extracellular free cholesterol accumulation, thus can lead to CCs formation (24). A rodent model study showed that after only 1 week of hyperlipidemia, CCs were formed and deposited in the plaque and increased with prolonged length of a high-fat diet treatment (20). We postulate that blood lipid in patients with CCs is higher than in patients without CCs. However, in our study, no significant differences were observed between patients with CCs and patients with non-CCs in blood lipid profiles except Lp(a). An multivariate analysis showed that blood lipid profiles were not independent predictors of CCs. Our results were consistent with previous studies, which showed that LDL-C and HDL-C levels were comparable between the CCs group and the non-CCs group (7, 10). The reason for this may be that except for overloaded low-density lipoprotein or impaired HDL function, many local physical changes such as saturation, temperature, hydration, and PH can enhance cholesterol crystallization (25).

Cholesterol crystals have been identified as a major catalyst for plaque vulnerability and as a potential biomarker for atherosclerosis. Abela et al. examined plaque samples by using light microscopy and SEM and found that the presence of CCs was strongly associated with plaque rupture and/or erosion (26). Nishimura et al. investigated CCs in patients with SAP and ACS and found that CCs within the culprit lesions became more frequent with the increase of the features of plaque vulnerability (10). Fujiyoshi et al. demonstrated that the presence of CCs was associated with a higher prevalence of vulnerable characteristics and was significant with a higher rate of 1-year major adverse cardiovascular events (MACEs) (7). Katayama et al. revealed that CCs were more frequently found in AMI patients with plaque rupture than in AMI patients without plaque rupture (27). The presence of CCs invading fibrous caps were an independent risk factor for plaque rupture except for rich lipid and thin fibrous caps (27). Our results showed that the culprit lesions with CCs in patients with ACS had more characteristics (TCFA, layered plaque, thrombus, accumulation of macrophages, plaque rupture, micro-channel, calcification, less MLA, and longer lesion length) of vulnerable plaque. In turn, TCFA, layered plaque, MLA, macrophage accumulation, and micro-channel were independently associated with CCs.

Mechanisms of CCs-inducing plaque vulnerability had been explored. Studies by Abela et al. showed that during the crystallization, CCs could perforate the plaque cap, thus leading to plaque rupture and/or erosion and subsequent thrombus formation (26, 28, 29). In addition, CCs could initiate inflammation *via* activating NLRP3-dependent inflammasome, driving plaque progression, and plaque instability. Studies using cell and animal models had showed that CCs prepared *in vitro* could activate NLRP3 inflammasome with the release of pro-inflammatory cytokines (30, 31). The expression of NLRP3 signaling pathway components including NLRP3, IL-1β, and IL-18 was documented to be associated with plaque vulnerability *in vivo* and *in vitro* studies (16, 32). Our study, for the first time, demonstrated the relationship between CCs in culprit lesions and NLRP3 expression in the human body. ACS patients with CCs in culprit lesions had higher expression of NLRP3 in macrophages and higher levels of IL-1β and IL-18 in serum than patients with non-CCs. Multivariate analysis showed that the levels of NLRP3 expression, IL-1β, and IL-18 were independently associated with CCs. Our results indicated that the NLRP3 pathway might be involved in CCs-inducing plaque vulnerability. In addition, no significant differences were observed between patients with STEMI, NSTEMI, and UA with regard to the NLRP3 expression and levels of serum IL-1β and IL-18. Our findings were in accordance with the results of the study by Altaf et al. (33), indicating that NLRP3 inflammasome and downstream cytokines might not be efficient enough to sense cell injury.

Recognizing the relationship between increased inflammatory status, CCs in coronary arteries, and plaque vulnerability could help to identify the high-risk plaques occurring in “vulnerable” patients and provide a new potential therapeutic target for these patients. Recent clinical studies demonstrated the efficacy of anti-inflammatory drugs in patients with CAD. CANTOS study showed that anti-inflammatory therapy targeting the IL-1β with canakinumab in patients with a stable coronary disease could lead to a 15% lower risk of cardiovascular events than was observed with placebo, but also led to a slightly higher incidence of fatal infections (34). The COLCOT Trial evaluated the effects of colchicine, a potent anti-inflammatory medication that possibly had effects on cellular adhesion molecules, inflammatory chemokines, and the inflammasome, on cardiovascular outcomes as well as its long-term safety profile in patients with recent (within 30 days) myocardial infarction, and demonstrated that colchicine at a dose of 0.5 mg daily led to a significantly lower risk of ischemic cardiovascular events than placebo. There were no significant differences in serious adverse events among the two groups, except for a higher rate of pneumonia in the colchicine group (0.9%) than that in the placebo group (0.4%) (35). Further studies are needed to investigate the effects of anti-inflammatory drugs in patients with CAD who exhibits CCs and increased inflammatory status.

## Limitations

There were some limitations in this study. First, data were acquired from a single center involving only patients with ACS. There was no control group consisting of stable patients with similar intracoronary imaging data available. Second, blood sampling in our study was collected from peripheral blood. Sampling from coronary veins and coronary culprit lesions has been shown to be more direct in comparison between biomarkers and coronary plaque characteristics/clinical presentation (36, 37). Markers of inflammation from venous blood might originate from different locations where inflammation could take place and inflammation of various origins might overstate the values of these indicators. Third, we only examined the plaque characteristics in culprit lesions using OCT; we did not examine the CCs distribution in non-culprit lesions. Fourth, the factors associated with NLRP3, IL-1β, and IL-18 were not explored in our study. Fifth, whether inflammatory markers would increase constantly and plaque remained unstable over time are still not known. Sixth, whether the CCs in culprit lesions could influence the clinical outcome in patients with ACS is still unclear.

## Conclusion

Patients with ACS with CCs in culprit lesions had higher expression of NLRP3, IL-1β, and IL-18, and had more vulnerable plaque characteristics than patients without CCs. In patients with ACS, CCs may interact with NLRP3 inflammasome activation, contributing to plaque vulnerability in culprit lesions.

## Data availability statement

The original contributions presented in the study are included in the article/supplementary material, further inquiries can be directed to the corresponding authors.

## Ethics statement

The studies involving human participants were reviewed and approved by Shanghai Ninth People's Hospital Ethics Committee of the institution. The patients/participants provided their written informed consent to participate in this study.

## Author contributions

CX participated in study design, data analysis, interpretation of results, and drafting of the manuscript. QC and LB participated in the interpretation of the results. ZY, ZX, HZ, QZ, JZ, and CW participated in the acquisition of data. RD and LF participated in the conception of the study, revision, and final approval of the manuscript. All authors read and approved the final manuscript.

## Funding

This work was supported by the Fundamental Research Program funding of Ninth People's Hospital affiliated with Shanghai Jiao Tong University School of Medicine (No. JYZZ010); The Scientific and Technological Foundation Projects of Shanghai Jiao Tong University, School of Medicine (No. 13XJ10050); and the National Natural Science Foundation of China (No. 81970330).

## Conflict of interest

The authors declare that the research was conducted in the absence of any commercial or financial relationships that could be construed as a potential conflict of interest.

## Publisher's note

All claims expressed in this article are solely those of the authors and do not necessarily represent those of their affiliated organizations, or those of the publisher, the editors and the reviewers. Any product that may be evaluated in this article, or claim that may be made by its manufacturer, is not guaranteed or endorsed by the publisher.
